# Nano Drug Delivery System for Tumor Immunotherapy: Next-Generation Therapeutics

**DOI:** 10.3389/fonc.2022.864301

**Published:** 2022-05-19

**Authors:** Lili Zhou, Manshu Zou, Yilin Xu, Peng Lin, Chang Lei, Xinhua Xia

**Affiliations:** ^1^ School of Pharmacy, Hunan University of Chinese Medicine, Changsha, China; ^2^ Institute of Innovation and Applied Research in Chinese Medicine, Hunan University of Chinese Medicine, Changsha, China

**Keywords:** tumor immunotherapy, nanotechnology, tumor microenvironment, drug delivery, nanomedicine

## Abstract

Tumor immunotherapy is an artificial stimulation of the immune system to enhance anti-cancer response. It has become a powerful clinical strategy for treating cancer. The number of immunotherapy drug approvals has been increasing in recent years, and many treatments are in clinical and preclinical stages. Despite this progress, the special tumor heterogeneity and immunosuppressive microenvironment of solid tumors made immunotherapy in the majority of cancer cases difficult. Therefore, understanding how to improve the intratumoral enrichment degree and the response rate of various immunotherapy drugs is key to improve efficacy and control adverse reactions. With the development of materials science and nanotechnology, advanced biomaterials such as nanoparticle and drug delivery systems like T-cell delivery therapy can improve effectiveness of immunotherapy while reducing the toxic side effects on non-target cells, which offers innovative ideas for improving immunity therapeutic effectiveness. In this review, we discuss the mechanism of tumor cell immune escape and focus on current immunotherapy (such as cytokine immunotherapy, therapeutic monoclonal antibody immunotherapy, PD-1/PD-L1 therapy, CAR-T therapy, tumor vaccine, oncolytic virus, and other new types of immunity) and its challenges as well as the latest nanotechnology (such as bionic nanoparticles, self-assembled nanoparticles, deformable nanoparticles, photothermal effect nanoparticles, stimuli-responsive nanoparticles, and other types) applications in cancer immunotherapy.

## Introduction

Currently, tumors are the second leading cause of death in the world after cardiovascular disease and the number of patients with cancer is still increasing (1). Cancer is now widely recognized as a global problem that lacks a global solution (2). The current anti-cancer interventions (surgical resection, chemotherapy, radiotherapy, and targeted drugs) have several therapeutic effects in the clinics (3). The available treatment regimens for cancer have made outstanding contributions in prolonging the survival time of cancer patients and improving the quality of life of patients in advanced stages (4). However, there are still severe challenges in the cure of most cancers and the improvement of its curative effect is still greatly limited. For instance, surgical removal of tumors is limited to the early stage of cancer, yet many cancers cannot be detected at the early stage (5). This method has an extremely poor prognosis for advanced cancers. The medical treatment will not only be easy to promote tumor metastasis but also cause damage to body functions (6). On the other hand, chemotherapy and radiotherapy are not site specific and may cause side effects (7). These therapies can damage normal body tissues while fighting the tumors (8). Although the targeted drug therapy can alleviate the adverse effects of drugs, it also suffers from tumor drug resistance, which often leads to tumor recurrence (9).

With the advancement in molecular and tumor biology, immunotherapy has become a new paradigm of clinical cancer treatment (10). In contrast to conventional treatment, tumor immunotherapy mainly targets immune cells (11). It activates the body’s immune system by inhibiting negative immune regulatory factors and enhancing the ability of immune cells to recognize tumor cell surface antigens to eliminate tumor cells (12, 13). It has been reported that immunotherapy possesses various advantages such as better efficacy, minimal side effects, and prevention of cancers from recurring (14). In recent years, with the deep understanding of tumor immune escape mechanisms, various new immunotherapies have been consecutively developed. From the launch of the first tumor immunotherapy drug (IFN-α) to the current immune checkpoint inhibitors (PD-1/PD-L1, CLTA-4) (15), CAR-T cell therapy (16), tumor vaccines (17), oncolytic viruses (18), and other new drugs, these drugs have shown good therapeutic effects and hence are approved for tumor immunotherapy.

Despite these major advances, the clinical use of immunotherapies faces several challenges related to both efficacy and safety. First, tumor cells have a lot in common with normal cells, making it difficult for the human immune system to distinguish them correctly. For example, CAR-T cells may attack not only tumor but also normal cells, resulting in “on-target off-tumor” toxicity (19). Second, due to the immunosuppressive microenvironment of solid tumors, some immune cells or cytokines infused intravenously cannot successfully reach the tumor site, resulting in immunotherapy being effective in hematological tumors, but still unable to overcome solid tumors (20). Nanomedicines can overcome some of the shortcomings of simple immunotherapy and enhance the effect of tumor immunotherapy. Moreover, nanomedicine gained much attention as one of the new technologies for diagnosis, treatment, and prevention of tumors as well as immune diseases (21). Nanomaterials refers to materials with a spatial size between 1 and 100 nm. Due to their specific spatial size, nanomaterials have different characteristics from other macroscopic materials, such as surface effects and small size effects (22). Compared with traditional drugs, nano drug delivery systems showed many advantages, such as better solubility and bioavailability, less toxic side effects, and the ability to pass through the blood-brain barrier (BBB) (23). Scientists first proposed the concept of liposomes in the 1960s (24) and after 30 years of research, the first nano-drug liposome, Adriamycin, was approved by the US FDA for marketing in 1995 (25). The main advantage of nano drug carriers is to target drugs to a specific site and enhance drug efficacy (24). Based on the route of administration (26), nano-targeted drug delivery systems are classified into intravenous administration, intramuscular and subcutaneous injection, oral administration, nasal administration, and transdermal administration as well as ocular administration. According to methods of action (26) nano-targeted drug delivery systems can be divided into: (1) Passive targeted preparations where the drugs are transported to target organs (liver, spleen, or lungs) through normal physiological processes with particles (emulsions, liposomes, microcapsules, or microspheres) as carriers; (2) active targeting agents refer to drug particles whose surfaces have been modified, which are not recognized by the mononuclear phagocytic system. The agents can also have special ligands attached to them to enable them to bind to the receptors of the target cells or organs; (3) physical and chemical targeting agents are those that direct particles to specific locations under the action of external forces such as temperature, pH, or magnetic field.

Anti-tumor immunotherapy mainly relies on regulating or activating the immune system of the patient. It efficiently inhibits or kills tumors with low toxicity (27). However, some clinical challenges of tumor immunotherapy still exist, such as the similarity of both tumor and normal cells makes it difficult for the human immune system to distinguish them correctly (28). For example (29), when using chimeric antigen receptor T-cell immunotherapy to attack tumor cells, it will be difficult to control “off-target effect” (attacks on other normal cells). Second, some immune cells or cytokines injected through the vein cannot reach the tumor site successfully, due to the immunosuppressive microenvironment of solid tumors. This leads to significant effects of immunotherapy on hematological tumors, but it is always unable to overcome the solid tumors (30).

With the development of materials science and nanotechnology, nanomedicine has shown several advantages expected to improve the effectiveness of immunotherapy. First, the nanomedicine can enhance the drug accumulation at the tumor site due to its unique high permeability and long retention (EPR) effect, thereby improving the therapeutic effect (31). Second, some nanomaterials have special properties, such as temperature-sensitive properties (32) and pH-sensitive properties (33). Drugs are modified through nanotechnology to achieve active and passive dual targeting. Enhanced drug targeting can reduce drug concentration in normal tissues or cells and improve treatment tolerance. Moreover, by optimizing the size and surface properties of the nano-carrier particles, their half-life in the reticuloendothelial circulation system can be prolonged to prevent rapid elimination by the system (34). Therefore, it is evident that the use of nanotechnology can overcome some of the shortcomings of immunotherapy and enhance the effect of tumor immunotherapy as well as provide more effective treatment options.

## Tumor Immune Escape Mechanisms

When tumor cells invade healthy tissues of the body, the immune system usually can recognize and eliminate them based on the tumor-associated antigens (TAAs) expressed on their surface. However, tumor cells can suppress the immune system of the host through a variety of mechanisms to evade the body’s immune system (35). Tumor immune escape mechanisms mainly include the following: (1) Down-regulates the expression of tumor cell surface antigens to reduce its immunogenicity, so that it cannot effectively activate the immune system (36); (2) up-regulates the expression of immune checkpoints on the cell surface (such as PD-L1) to inhibit the activity of T-lymphocytes, thereby evading the body’s immune system (37); (3) by recruiting immunosuppressive cells, myeloid-derived suppressor cells (MDSC), and regulatory T-cells (Treg) into the tumor immune microenvironment and secreting cytokines, it inhibits the immune response of the body to the tumor cells (38); and (4) inhibits the activity of immune cells in the tumor microenvironment by releasing acidic and toxic metabolites, thereby achieving immune escape (39). The immune system can detect and eliminate the tumor cells from the body. Tumor immunotherapy mainly kills tumor cells by reactivating the anti-tumor immune response of the body. Early tumor immunotherapy mainly used cytokines produced by the immune cells to directly attack the tumor cells, such as IL-2 and IFN. Subsequently, some new immunotherapies, such as immune checkpoint inhibitors, cellular immunotherapy, oncolytic viruses, and tumor vaccines, have gradually developed into the main force of tumor immunotherapy.

## Classes of Cancer Immunotherapy

### Cytokines

Cytokines are natural immune modulators secreted by a variety of immune cells such as lymphocytes, monocytes, and macrophages which regulates the immune response of the human body (40). The immunosuppressive cells (MDSC and Treg) recruited by the tumor tissues can secrete inhibitory cytokines, suppress the body’s immune system, and hence achieve immune escape. On the contrary, stimulating cytokines can activate the immune system and kill tumor cells. Therefore, stimulatory cytokines delivered to the tumor site can be activated to realize the treatment of the tumor in patients with cancer.

Stimulatory cytokines are the earliest drugs used in tumor immunotherapy. The most widely used clinically stimulating cytokine drugs are INF-α and IL-2 (41). Interferon-α (INF-α) can enhance the activity of dendritic cells (DC) and natural killer cells (NK) hence improve the body’s anti-tumor ability (42). Specifically, INF-α is a representative FDA-approved cytokine which has been used in the clinic to treat leukemia since 1986 (43). Interferon is usually produced by immune cells in response to microbial pathogens. They induce an immune response by stimulating maturity of a large number of immune cells including macrophages, natural killer (NK) cells, lymphocytes, and dendritic cells (44). On the other hand, interleukins are mainly secreted by CD4+ helper T-cell subsets and participate in the activation of CD8^+^ cytotoxic T-cells, macrophages, and NK cells (45).

In 1992, recombinant human interleukin-2 (rhIL-2), produced by genetic engineering technology, was approved for the treatment of kidney cancer whereas it was approved for the treatment of metastatic melanoma in 1998 (46). Three other immune-activating recombinant cytokines (IFN-α2b, IFN-α2a, and IL-2) have been approved for cancer immunotherapy (47). Furthermore, several other cytokines, including IL-17 and IL-15, are under clinical development (48). However, due to the short half-life of the cytokines, the treatment method generally adopts high-dose therapy to cause rapid injection of vascular leakage and cytokine release syndrome. In addition, cytokine therapy can promote the survival of regulatory T-cells, induce the death of stimulated T cells, and ultimately lead to autoimmune attacks on healthy tissues (49).

### Checkpoint Inhibitors

Immune checkpoints are signal pathways on the surface of T-cells that inhibit activation of T-cells and hence actively participate in immune responses (50). If the immune checkpoint is activated, it suppresses the immune cells. To avoid the attack of the immune system, cancer cells generally activate immune checkpoints to inhibit the immune system from attacking them. In addition, if the activation of immune checkpoints by cancer cells is prevented, the immune system can maintain normal functions of attacking and killing cancer cells. In 2018 (51), the Nobel Prize was awarded for the identification of immune checkpoints. This great achievement has led to the development of antibodies targeting these checkpoints for anticancer therapy. Currently, more than 10 kinds of immune checkpoints have been developed, of which the most widely studied immune checkpoints are CTLA-4 (Cytotoxic T-lymphocyte antigen-4) and PD-1/PDL-1 (52).

CTLA-4 is the first negative regulator found to activate T cells (53). CTLA-4 and CD28 belong to the B7 family of receptors, providing feedback on the early stages of negative and positive immune responses in the process of T-cell activation. However, the affinity of CTLA-4 for both ligands is about 100-fold higher than that of CD28 (54). In the lymph node, CTLA-4 outcompetes the stimulatory receptor CD28 for binding to T-cell ligands CD80 and CD86, which inhibit T-cell activity and thus promote tumor progression. The T-cells remain active by blocking the interaction between these ligands and CTLA4, and hence can recognize and kill tumor cells. After many clinical trials, the FDA approved the first antibody drug ipilimumab for the immune checkpoint CTLA-4 in 2011 (55). Ipilimumab is an antibody drug mainly used for the treatment of melanoma, which can improve the survival of patients by 1 to 2 years.

Programmed cell death-1 (PD-1) and PD-L1 blockade represent another major family of checkpoint inhibitors. PD-1 is expressed on activated T-cells, which enables T-cells to recognize abnormal and cancerous cells. To avoid the recognition and elimination of T-cells, tumor cells express PD-L1 which binds to PD-1 on T-cells and inactivates the T-cells (56). Inhibiting the interaction of PD-1 and PD-L1 results in significant enhancement of T-cell function. The first PD-1 inhibitor pembrolizumab was approved by the FDA in 2014 for the treatment of melanoma and lung cancer whereas the first PD-L1 inhibitor, atezolizumab for the treatment of bladder cancer was approved in 2016 (57, 58). Checkpoint inhibitors may have severe side effects in numerous organs (skin, gastrointestinal tract, endocrine tissues, and liver), and different tumor microenvironments have distinct mechanisms of immunosuppression (59).

### Adoptive Cell Transfer

Adoptive cellular immunotherapy refers to a method of passive immunotherapy in which immune cells with tumor-killing effects are expanded, cultured *in vitro*, and then returned to tumor patients to achieve the purpose of anti-tumor (60). Adoptive cell transfer therapy (ACT therapy) includes tumor infiltrating T cell (TIL) therapy, chimeric antigen receptor T cell therapy (CAR-T), and T cell receptor therapy (TCR).

Adoptive cell transfer therapy requires immune cells differentiated from patients, donors, or stem cells. These immune cells are then activated, expanded *in vitro*, genetically modified, and then injected into the patient through a peripheral vein or local artery.

Tumor infiltrating T-cells (TILs) are the lymphocytes that accumulate at the tumor margins or infiltrate within the tumor. They are not a homologous population of lymphocytes, whose function was first described in detail by Klein et al. (61). Among tumors infiltrating lymphocytes, melanoma is the first example of tumor-reactive T cells that can be expanded *in vitro* for adoptive transfer. In 1988, Rosenberg et al. conducted the first clinical trial of ACT using TILs at NIH (62). The standard method of exposing tumor-derived lymphocytes to high dose IL-2 *in vitro* followed by rapid expansion in a mixed feeder population, was found to be an effective treatment option for patients with refractory metastatic melanoma.

Chimeric antigen receptor T-cells (CAR-T) and TCR therapies are based on the same principle. They both extract T-cells from the patient’s peripheral blood and genetically engineer them to express chimeric antigen receptors (CAR) or new T-cells that can recognize cancer cells. T cell receptor (TCR) activates and guides T-cells to kill cancer cells. On the other hand, CAR-T uses exogenous gene transfection technology to express the fusion protein of single-chain variable fragment (scFv) that recognizes tumor-associated antigens and T-cell activation sequence on the surface of T-cells (63). Upon injection, CAR T-cells recognize the targeted antigen on tumor cells to induce tumor cell death. CD19 (present on both benign and malignant T-cells) is an ideal target of CARs (64). The CD19 targeted CAR-T have been tested and shown to be effective in large clinical trials. In 2017, the FDA approved CAR-T therapy, Tisagenlecleucel, for treatment of acute lymphocytic leukemia (65). Subsequently, another CAR-T immunotherapy, Axicabta-gene ciloleucel, was approved by the FDA for the treatment of diffuse large B-cell lymphoma in the same year (66).

Although CAR-T therapy currently shows significant efficacy advantages in the treatment of hematoma and lymphoma, it also exhibits some risks in the process of clinical use. The cytokine release syndrome (CRS) and immune effector cell-related neurotoxicity syndrome (ICANS) are the most common adverse reactions of CAR-T cell immunotherapy (3). They often manifest as fever, hypotension, and hypoxia as well as capillary symptoms such as blood vessel leakage and impaired cognitive ability. To substantially improve the clinical efficacy of adoptive cell immunotherapy (ACT) against solid tumors, researchers might need to look closer into recent developments in the other branches of adoptive immunotherapy (67).

### Cancer Vaccines

Cancer vaccine is one of the hotspots of research in recent years. It can use tumor-associated antigens (TAAs) such as DNA, RNA, protein, or peptides to regulate the immune system. The introduction of tumor vaccines into patients can overcome the immunosuppressive state caused by tumors. This enhances immunogenicity, activates the patient’s immune system, and induces cellular and humoral immune responses to achieve the goal of controlling or eliminating tumors. After entry of the vaccine into the human body, the antigen recognizes it using the pattern recognition receptors (PRRs) on the surface of antigen-presenting cells (APCs). The PRRs are macrophages or dendritic cells (DCs) that exist in the periphery and are transported to the draining lymph nodes or spleen immune organs. In 2010, the FDA approved sipuleucel-T (Provenge) for the treatment of advanced prostate cancer. Provenge was hence the first autologous active immunotherapy drug and the first true therapeutic cancer vaccine (68).

The anti-tumor mechanisms of tumor vaccines include (69) activation of the immune system, activation of T-cells, induction of endogenous antigen-specific cytotoxic T-lymphocyte response, and secretion of cytokines.

Currently, tumor vaccines are on the market and under development. They can be divided into four categories: (1) whole-cell vaccines (70). They are further divided into tumor cell vaccines and dendritic cell (DC) vaccines according to the source of the cells. Tumor whole-cell vaccines have their unique advantages whereas tumor-specific antigens are not yet clear. Tumor whole-cell vaccine contains a full range of tumor-associated antigens (TAA) and is rich in CD8^+^ T cells and CD4 helper T-cell epitopes. They can also express MHC class I and class II restricted antigens, causing a comprehensive and effective anti-tumor response as well as inducing the growth of effective memory T-cells.

(2) Tumor peptide vaccines. They trigger T cell responses through dendritic cells (DCS) (70). The polypeptide vaccines prepared using antigen polypeptides eluted from the surface of tumor cells or proteins abnormally expressed inside tumor cells have the advantages of strong specificity and high safety. Polypeptide vaccine is one of the most widely researched and applied tumor vaccines.

(3) Genetic engineering vaccines. The tumor-related antigen peptides in the vaccine can be specifically recognized by T-cells. This characteristic can cause the immune system of the host to react actively and destroy tumor tissues. It provides safety, strong specificity, and other characteristics. A variety of tumor vaccines currently on the market (such as HPV and M-Vmax) are genetic engineering vaccines (71). They use genetic engineering technology to load genes encoding tumor-specific antigens onto recombinant viral vectors or plasmid DNA and inject them directly into the human body.

With the help of the vector itself or the human gene expression system, a genetic engineering vaccine can continue to cause specific humoral immunity and cellular immunity. This is an incomparable advantage of genetic engineering vaccines over the other tumor vaccines. Therefore, these vaccines have drawn a lot of attention in tumor biotherapy research. Previous studies have shown that fusion of DNA encoding cytokines and bacterial proteins with plasmid DNA of genetic engineering vaccines can effectively improve its immunogenicity and cause a strong immune response to antibody tumor vaccines (17).

(4) Monoclonal antibody tumor vaccine. It is designed based on antibody cell-mediated cytotoxicity (ADCC) theory. The monoclonal antibody can highly specifically bind to the corresponding antigen and has a good molecular targeting function. Currently, there are two types of monoclonal anti-tumor drugs: one is anti-tumor monoclonal antibodies; the other is anti-tumor monoclonal antibody conjugates or immunoconjugates. Monoclonal antibody drugs combine with tumor antigens to co-stimulate DC and stimulate CD8^+^ T-cells. This technology has hence allowed significant progress in the treatment of melanoma and breast cancer.

## Cellular Targets of Nano Preparations for Immunotherapy

Dendritic cells (DC) are the most important professional antigen presenting cells, which have the unique ability to maintain autoantigen tolerance and induce primary immune responses. The application of nanotechnology to tumor treatment can improve the delivery efficiency of antigens and the efficiency of dendritic cell activation. The cross-presentation of dendritic cells and the further cooperation of immune cells can stimulate a strong immune response and effectively inhibit tumor growth. Some nanocarriers also act as good immune adjuvants, which can synergize with antigens to produce stronger immune responses.

**Figure 1:**
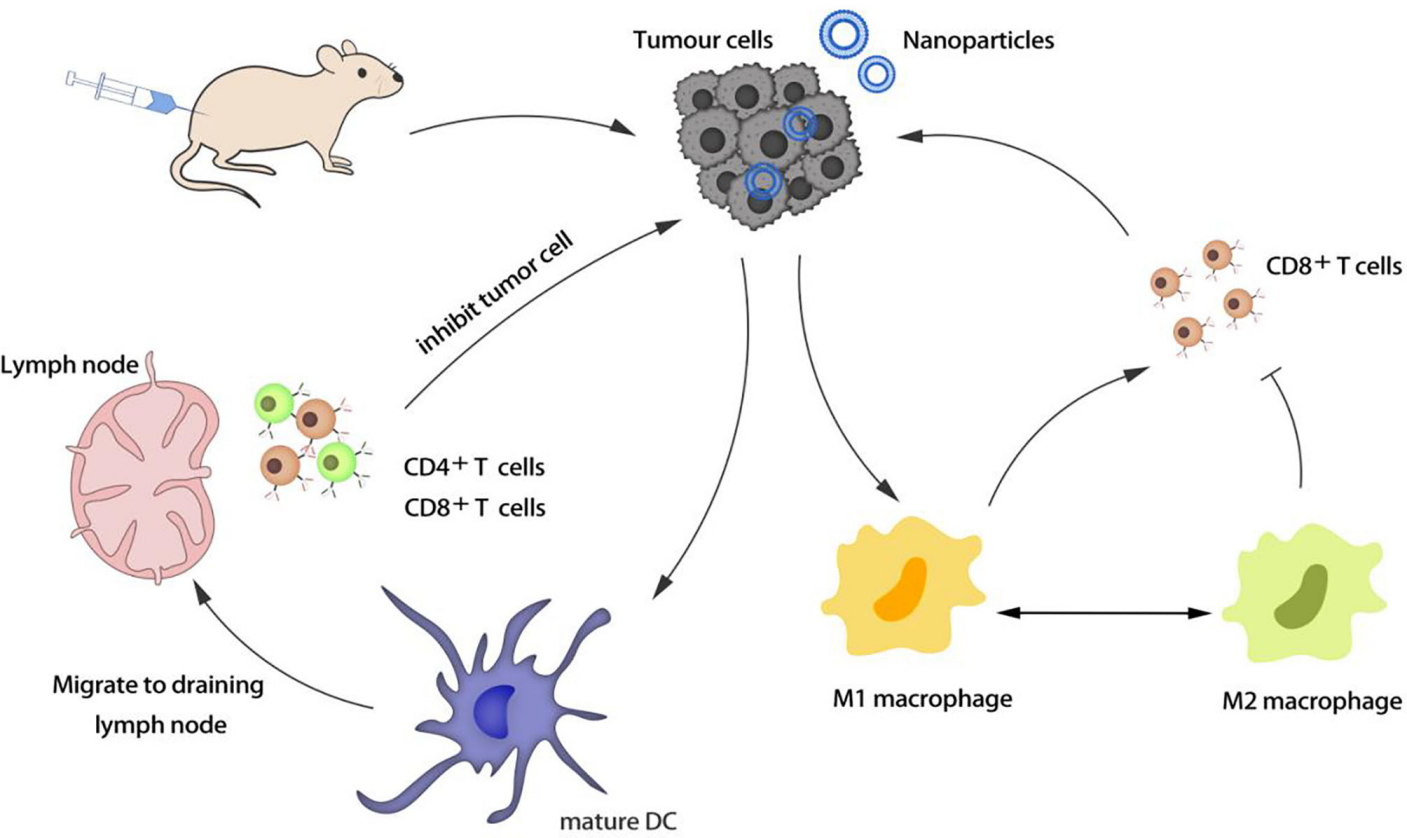
Activation and regulation of antigen presenting cells (APCs).

Macrophages are important phagocytes and antigen-presenting cells, which are used to regulate inflammation. M1 macrophages can resist pathogens and kill cancer cells, while M2 macrophages can promote the growth and invasion of cancer cells. Enhancing the enrichment of macrophages in tumors and regulating the polarization of macrophages from M2 to M1 are new strategies to enhance immunotherapy (**Figure 1**).

**Figure 2:**
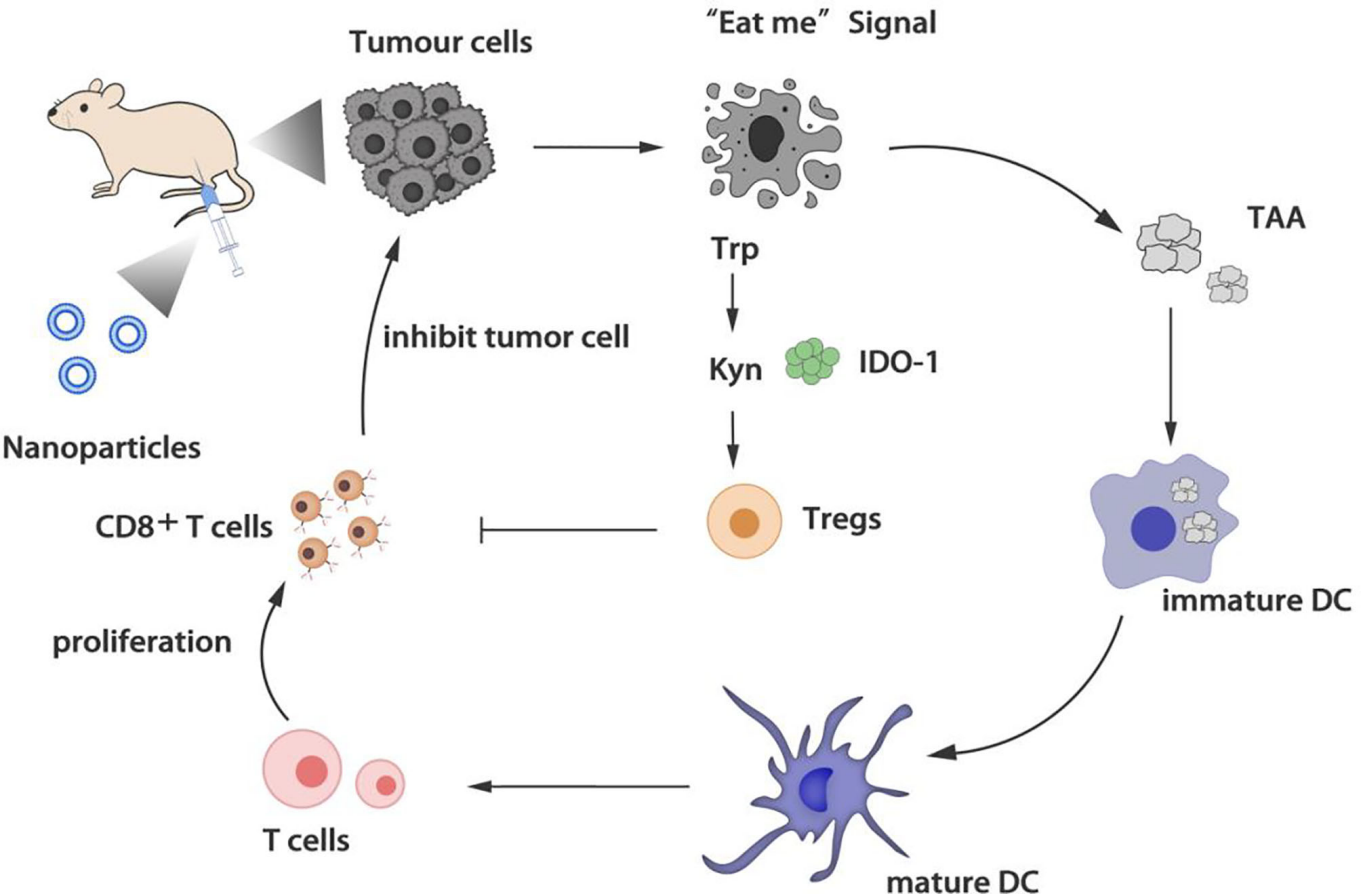
Regulation of T cells.

The activation of T cells is the core of the immune response. Many regulatory T cells (Tregs) accumulated in the tumor microenvironment can inhibit the anti-tumor immunity of cytotoxic T lymphocytes (CTLs). Choosing a nano-delivery system to target the tumor site and down-regulating regulatory T cells is beneficial to reverse the tumor immunosuppressive microenvironment (**Figure 2**).

**Figure 3:**
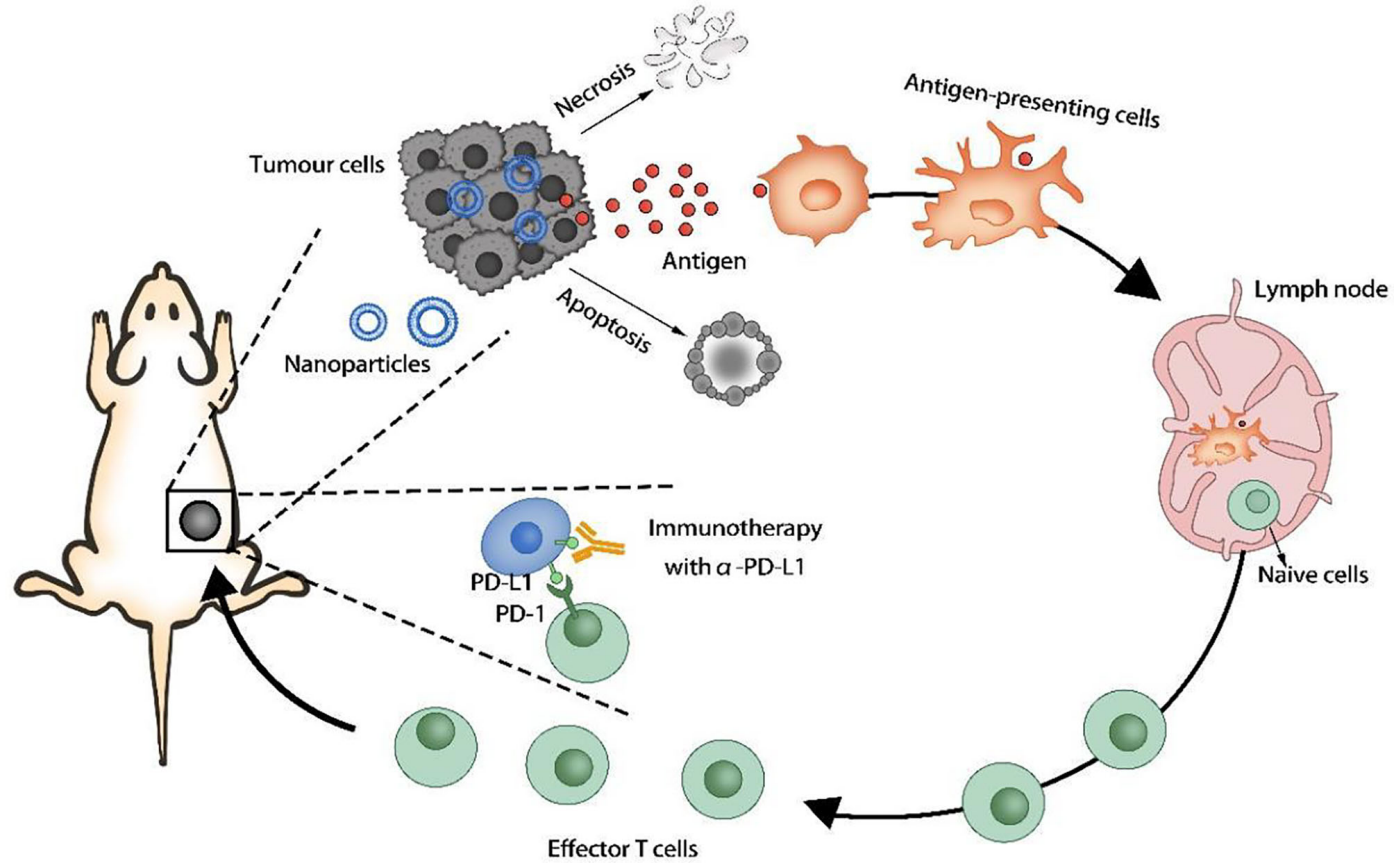
Induction of immunogenic cell death (ICD).

Tumor cells can overexpress certain proteins or receptors that interact with immune cells, thereby blocking immune activity and causing tumors to evade immune surveillance. The programmed cell death 1 ligand 1 (PD-L1) on the tumor cell membrane can inhibit the immune function of T cells, and the CD47 protein can prevent the phagocytosis of tumor cells by macrophages and dendritic cells. The use of nanocarriers to deliver inhibitors of the above receptors or proteins to prevent immune escape of tumors can significantly improve the effect of immunotherapy. Some of the drugs encapsulated in nano-formulations not only can directly kill cancer cells, but also induce tumor immunogenic cell death (ICD) by generating reactive oxygen species (ROS) and induce immune responses (**Figure 3**).

## Nano Drug Delivery System for Tumor Immunotherapy

Nanoformulation is a drug delivery system prepared from polymers, lipids, inorganics, metals, and other materials. Drug-loaded nanoformulations can be retained at the tumor site through enhanced permeability and retention effects, enabling passive targeted drug delivery. Nano drug delivery system has the advantages of stabilizing the biological activity of protein and nucleic acid drugs, increasing the solubility of insoluble drugs, and improving the therapeutic index of drugs. They can effectively reduce the systemic toxicity of drugs and overcome the drug resistance of chemotherapeutic drugs, and have been widely used in the diagnosis, treatment, and prognosis of tumors. However, the special pathological structure of tumors and their inhibitory immune microenvironment limit the efficacy of drugs. The development of nanotechnology has provided a good research foundation for the development of functional nanoformulations. It is different from traditional tumor nano delivery systems that directly kill tumor cells. In immunotherapy, nano delivery systems deliver drugs or active molecules to tumor sites, causing immune responses directly or indirectly. In addition, through functional modification or structural modification of the nanocarrier, the off-target rate can be reduced, and the long circulation time and delivery efficiency in the body can be improved.

**Figure 4:**
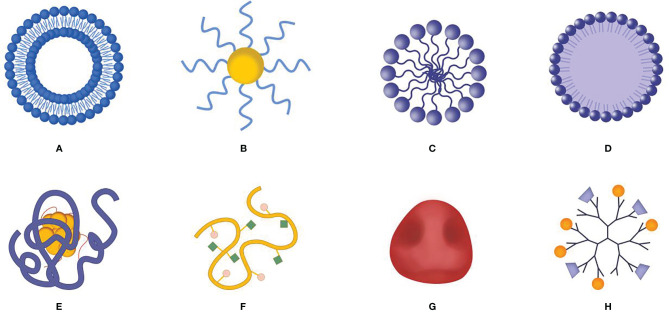
Schematic diagram of nano delivery system carrier. **(A)** Liposome, **(B)** gold nanoparticle, **(C)** polymeric micelle, **(D)** solid lipid nanoparticles, **(E)** protein and molecular complex, **(F)** polymer-molecular conjugates, **(G)** biofilm bionic nanoparticles, **(H)** dendrimer.

New nano-delivery systems (**Figure 4**) have made progress in regulating the immunosuppressive microenvironment (including releasing the influencing factors of immunosuppression, enhancing the intensity of autoimmune responses), remodeling tumor pathological structures that affect the efficacy of immunotherapy, and enhancing the efficacy of immunotherapy. Therefore, in-depth research on the role of novel nano-delivery systems in regulating the immunosuppressive tumor microenvironment, reducing the adverse reactions, and enhancing the efficacy of immunotherapy will provide guidance for the selection of therapeutic strategies for clinical tumor patients.

### Delivery of Tumor Antigens

The use of tumor cell-associated antigen (tumor-associated antigen, TAA) to reactivate response of the immune system of the body against tumor cells plays a significant role in early prevention and treatment of cancer (72, 73). Compared with direct injection of TAA, the use of nano-delivery vehicle can protect the antigen from degradation and target it to dendritic cells (DCs) or T-lymphocytes as well as produce the effect of cross-presenting the antigen. This effectively stimulates cytotoxic T-lymphocytes (cytotoxic lymphocyte L, CTL) and promotes anti-tumor immunity (74–76).

Muraoka et al. (77) developed a partially hydrophobized nanogel with long peptide antigen (LPA) inside by the modification with cholesteryl groups (CHP). More experiments have shown that the subcutaneous injection of CHP nanogel has a fast accumulation in the draining lymph nodes. They enter the lymph node medullary macrophages in a highly selective manner, thereby inducing a significant antigen-specific T cell response. Various findings indicate that the MUC4 does not only promote the progression of pancreatic cancer functionally, but also is a potential tumor antigen for pancreatic cancer immunotherapy (78–80). If the MUC4 protein is injected as a vaccine antigen, the injection of this recombinant protein provides the advantage of exposing immune cells to multiple epitopes compared with peptide vaccines.

Nano-vaccines are formed through encapsulation of recombinant MUC4β subunits into amphiphilic polyanhydride copolymers. The results of the formulation and characterization experiments showed that MUC4β was stably released and its structural integrity as well as antigenicity were maintained. Further, mice immunized with MUC4β-loaded nanoparticles generated MUC4β-specific antibody responses (81).

DCs are important antigen presenting cells (APCs) in the body. APCs are the key cells to trigger immune response. Modifying molecules that bind to specific cell type receptors on APC can enhance antigen uptake and subsequent T cell activation. Affandi et al. (82) demonstrated a liposome-based nano-vaccine carrier that uses ganglioside as a targeting ligand to deliver tumor antigens to human CD169/Siglec-1^+^ antigen-presenting cells. Through *in vitro* binding/uptake methods, it has been proven that ganglioside liposomes effectively bind to APC expressing CD169^+^, leading to strong cross-presentation and activation of tumor antigen-specific CD8^+^T cells. *In vivo* mice models have shown that the antigen targeting CD169^+^ macrophages cause a strong CD8^+^T cell activation response.

Zou et al. (83) constructed a mannose-modified poly(β-aminoester) (PBAE) pH-sensitive nano-vaccine that can co-deliver the tumor-associated antigen polypeptide Trp-2 and the TLR4 agonist monophosphate lipid A(MPLA). The PBAE immune vaccines have shown a strong ability to induce the maturation of DCs *in vitro* and *in vivo*. In addition, a combined therapy of the immune vaccine with PD-L1 can overcome the tumor immunosuppressive microenvironment, enhance tumor infiltrating CTLs, and achieve a synergistic anti-tumor effect.

The stimulating factor of interferon gene (STING) pathway plays a key role in initiating and spreading the endogenous mechanism of anti-tumor T cell immunity. A synthetic cancer nano-vaccine platform (nano-STING vax) has been developed in response to this approach. The platform loads cGAMP and peptide antigens into pH-responsive endosomolytic polymersomes (84). Endosoluble polymers loaded with cGAMP and peptide antigens have the ability of dual cytoplasmic delivery, which leads to DC activation and antigen presentation, thereby enhancing CD8^+^T cell activation. It has been found that a combination of activated CD8^+^T cells with immune checkpoint blockade inhibits tumor growth in a variety of mouse tumor models and even leads to complete tumor rejection and long-lasting anti-tumor immune memory. Most cancer vaccines only activate the Th2 pathway, and it is still difficult to activate the Th1 pathway to induce cytotoxic T-lymphocytes (CTL).

The strategy of delivering antigens to the draining lymph nodes (DLNs) to improve CTL activation has attracted the interest of immunology circles. Miura et al. (85) explored the ability of self-assembled nanogels (CHP) loaded with antigen (ovalbumin, OVA) to activate this pathway. The high colloidal stability and hydrophilic surface of CHP nanogels give it immune invisibility. This ability enables CHP nanogels to deliver antigens to lymph nodes and trigger humoral immunity as well as antibody induction through high cross-presentation on APCs.

Results of *in vivo* experiments showed that the CHP nanogel vaccine can successfully induce OVA-specific CTL and antibodies. Compared with OVA alone, the CHP nanogel vaccine has a good anti-tumor effect in OVA tumor-bearing mice. Inspired by the splenic antigen-presenting cell targeting capacity of senescent RBCs, Xiao et al. (86) developed an antigen delivery system based on red blood cell-derived nano-erythrocytes. The tumor antigens were loaded onto the nanoerythrosomes through fusing the tumor cell membrane-associated antigens with nanoerythrosomes. This tumor antigen-loaded nanoerythrosomes elicited antigen responses *in vivo*, and when nanoerythrosomes combined with anti-programmed death ligand 1 (PD-L1), it can inhibit tumor growth in B16F10 and 4T1 tumor models.

### Delivery of Immune Check Point Inhibitor Antibodies

Development of immune antibodies has revolutionized cancer immunotherapy. For example, monoclonal antibodies (mAbs), which have strong specific binding ability, can reduce adverse reactions at non-targeted sites, and inhibit the interaction between immune checkpoint molecules, such as programmed cell death 1(PD-1), and its ligands PD-L1 (87, 88). However, monoclonal antibodies need to be encapsulated in nanocarriers and delivered directly to the tumor microenvironment due to their poor pharmacokinetics, limited tumor penetration ability, and difficulty in crossing biological barriers (89). Liu et al. (90) developed an amphiphilic polymer micelle encapsulating anti-PD-L1 antibody and NIR-II photosensitizing dye, and achieved high efficacy in photodynamic therapy (PDT) as well as cancer therapy immune effects. After light irradiation, micelles induced formation of O_2_ to kill MC38 cancer cells, while normal cells were not damaged. Moreover, the MC38 tumor was removed after 30 days, and did not recur within 40 days, and tumors failed to grow in mice within 7 days of re-inoculation with cancer cells. Overall, these results indicated that the mice have a long-lasting immune memory, which can help protect them from the risk of cancer recurrence. Cancer cells can evade recognition by the immune system by up-regulating integrin-associated protein (CD47). Notably, blocking CD47 can activate phagocytes and promote antigen presentation.

Here, the researchers engineered an albumin-based nanoparticle that responds to reactive oxygen species (ROS). Its core comprises anti-PD-1 (aPD1) while the outer shell is anti-CD47 (aCD47) (aPD1@aCD47 complex), which are coupled *via* ROS reactive linker. In the ROS-rich tumor microenvironment, it was found that the complex sustainably releases CD47 to activate the innate immune system, thereby recognizing cancer cells and promoting responsive growth of T cells. Subsequent release of aPD1 effectively exacerbates the attack of alloreactive T cells on cancer cells *via* blocking PD1. A previous study, using a melanoma tumor model, demonstrated that a synergistic anti-tumor effect was achieved, and this was accompanied by an enhanced T cell immune response as well as a reduced immunosuppressive response (91). Accumulating evidence has shown that tumor immunotherapy is a continuous multi-step process (92, 93). Therefore, an ideal immunotherapy nanoformulation can simultaneously perform the three stages of the cancer immune cycle, including antigen presentation (stage 1), lymphocyte activation and proliferation/differentiation (stage 2), as well as tumor elimination (stage 3). In a previous study, researchers employed an assembly strategy to rationally design hyaluronidase-sensitive hyaluronic acid-Ce6 (HC), cathepsin-sensitive PLL-1-mt (PM), and anti-PD-L1 monoclonal antibodies (aPD-L1) as a PD-L1@HC/PM NPs (94). They found that the resulting PD-L1@HC/PM NPs caused a step-by-step detachment of antigens induced by the three components toward their on-demand target site for immunotherapy. Taken together, these findings affirmed that NPs are an excellent immunotherapeutic nanoplatform for simultaneous execution of the aforementioned three stages. Functionally, NPs can be used to fight tumor metastasis, recurrence, and postoperative regeneration, due to cascade amplification of the cancer-immune cycle. Apart from tumor immunotherapy, immunomodulatory monoclonal antibodies (mAbs) have been used to reactivate dysfunctional T lymphocytes. Another strategy involves promoting the combination of effector immune cells and tumor cells through chimeric antigen receptor (CAR) T cells or bispecific T cell binding antibodies (BITS). Combining the two strategies results in better efficacy compared to a single method alone. Results from a previous study showed that immobilizing two monoclonal antibodies targeting effector cells and tumor cells onto a single nanoparticle can achieve the functions of these two methods (95). Moreover, results from several tumor mouse models have indicated that nanoparticles combining the two methods have a significant effect on the anti-tumor immune response mediated by T cells, natural killer cells, and macrophages. Inspired by the inflammatory tropism of platelets, the researchers designed a platelet modified with anti-programmed death ligand 1 (aPDL1) and polylactic acid-glycolic acid coated with indocyanine green (PLGA-ICG) as model photothermal agent (96). Its evaluation in a triple-negative breast cancer (4T1) tumor-bearing mouse model revealed that antibody-conjugated platelets can effectively target tumors that are not completely ablated by thermal ablation (TA). Further analysis revealed that platelet activation could promote the transport of anti-PD-L1 antibody to the ablation area of the residual tumor, thus effectively inhibiting recurrence of local tumors and improving the survival rate.

### Delivery of mRNA Tumor Vaccine

Although efficacy of nucleic acid-based immunotherapy has been proven in preclinical studies, the expected outcomes from clinical trials have not been achieved (97, 98). One major limitation of this approach has been the lack of appropriate delivery systems to prevent degradation of nucleic acids, promote cellular uptake, and enable specific cell delivery (99, 100). In recent years, the development of biocompatible and cell-targeting nanomaterials has significantly improved efficacy of mRNA-based anti-tumor vaccines. Consequently, researchers have successively applied new nanomaterials to deliver nucleic acids, and have achieved tremendous progress (101). For example, Oberli et al. (102) formulated a lipid nanoparticle aimed at delivering mRNA vaccines to induce cytotoxic response in CD 8^+^T cells. Structurally, the nanoparticle comprised an ionizable lipid, a phospholipid, cholesterol, a polyethylene glycol (PEG) containing lipid, and an additive for delivery of mRNA vaccines. At low pH, the use of positively charged ionizable lipids to complex with the negatively charged mRNA enabled cellular uptake and endosomal escape. Moreover, the authors used an aggressive B16F10 melanoma model to demonstrate that mRNA encoding melanoma-related self-antigens, namely tyrosinase-related protein 2 (TRP2) and glycoprotein 100 (gp100), could overcome self-tolerance, thereby resulting in tumor shrinkage, and significantly prolonging the overall survival times of the mice. A recent study reported that systematic administration of an advanced hybrid lipopolymer shell mRNA nanoparticle could cause a strong cytolytic T cell response, thereby resulting in high anti-tumor efficacy (103). Moreover, a cationic liposome/protamine complex (LPC) was found to be a safe and effective vaccine delivery system for transnasal mRNA-encoded CK19 (mCK19) (LPC/mRNA) for cancer immunotherapy. Notably, this delivery system can promote antigen uptake in DC, effectively stimulating their maturation, promoting secretion of cytokines, and inducing anti-tumor immune responses. Furthermore, evidence from an aggressive Lewis lung cancer model revealed that LPC/mRNA could stimulate a strong cellular immune response and attenuate tumor growth in mice (104). Another research group developed a nanocapsule with a flexible polysaccharide shell and hollow core, termed Sugar-capsule, that was entirely composed of polysaccharides derived from the microbial cell wall. Their results indicated that the sugar capsules, which were composed of mannan (Mann-capsule) carrying messenger RNA (mRNA), promoted strong activation of DC and antigen presentation, and further induced powerful antigen-specific CD4 and CD8α T cell responses with anti-tumor efficacy *in vivo (* 105).

To solve the problem of low efficacy of single mRNA treatment, several groups have applied the combined treatment strategy, and found that it has potential to improve efficacy of clinical treatments and enhance immune resistance. An adjuvant pulsed mRNA vaccine nanoparticle (NP), loaded with both ovalbumin-coded mRNA and a palmitic acid-modified TLR7/8 agonist R848(C16-R848), was developed. Notably, co-delivery of nanoparticles significantly increased expansion of CD8 T cells and infiltrated the tumor bed, compared with nanoparticles encapsulating a single mRNA, thereby inducing an effective adaptive immune response (106). Moreover, the findings of Verbeke et al. (107) revealed that a nanoparticle platform, called mRNA Galsomes, successfully co-delivered nucleoside-modified antigen-encoding mRNA, as well as glycolipid antigen and immunopotentiator α-Galactosylceramide (α-GC) to antigen-presenting cells. Driven by invariant natural killer T cells (iNKT), mRNA Galsomes induced multifunctional innate and adaptive tumor-specific immune responses in mice. Although treatment of triple negative breast cancer (TNBC) patients has remained a huge clinical challenge, tumor vaccines based on tumor-associated antigens represent a promising treatment strategy. For example, researchers have constructed an NP that combines an anti-cytotoxic T lymphocyte-associated protein 4 (anti-CTLA-4) monoclonal antibody and mRNA encoding tumor antigen MUC1 for enhancing anti-tumor benefits. *In vivo* studies demonstrated that the NPs can successfully express tumor antigens in DC in the lymph nodes, as well as activate and amplify tumor-specific T cells. Notably, a combination of immunotherapy using the vaccine and anti-CTLA-4 monoclonal antibody was found to significantly enhance anti-tumor immune response, relative to mRNA or monoclonal antibody therapies alone (108).

### Delivery of Immunologically Active Cytokines

Direct modification of immunocompetent molecules on the surface of nanoparticles or wrapping them inside nanoparticles is another effective means for achieving tumor immunotherapy, aside from inducing immune cells to release inflammatory factors. For example, Gasparri et al. (109) recently used a nano-gold carrier for targeted delivery of recombinant interleukin IL-12, and achieved favorable anti-tumor efficacy. Previous studies have demonstrated that low doses of paclitaxel can enhance the therapeutic effect of cytokines by inhibiting activity of regulatory cells (Treg) (110). Song et al. (111) constructed a tumor microenvironment-sensitive erythrocyte membrane-coated nanogel, and simultaneously loaded it with paclitaxel and cytokine IL-2. Paclitaxel exerts an immunomodulatory effect, activates DC, reduces the number of Tregs, and further enhances activation of immune effector cells induced by small doses of IL-2. The developed nanogel was found to improve penetration of drugs at tumor sites, while its mediated immunotherapy combined with chemotherapy exhibited anti-tumor synergistic effects.

The main requirement for immunotherapy is to directly interfere with specific immune cells *in vivo*. However, targeting of T cells for biomedical applications remains an obstacle owing to their low endocytic activities. In a previous study, researchers coupled IL-2 to the surface of hydroxyethyl starch nanocapsules, they demonstrated specific T cell targeting *in vitro* and *in vivo* by IL-2 receptor-mediated internalization (112). In addition, Cytimmune Sciences of the United States co-modified recombinant human tumor necrosis factor (rh TNF)-α and sulfhydryl PEG on the surface of gold nanoparticles, and successfully constructed a highly effective anti-tumor drug (113). Previous studies have shown that the nano-gold-TNF complex can significantly prevent uptake of a reticuloendothelial system (RES) and can be quickly targeted to tumor tissues within 4 h of administration. Notably, nano-gold-loaded TNF generate better anti-tumor activity and lower adverse reactions at lower doses compared to free TNF.

### Delivery of Natural Polysaccharides

Polysaccharides, such as lentinan and Ganoderma lucidum glycopeptide, have long been widely used in clinics as auxiliary drugs for improving body immunity (114, 115). Numerous studies have shown that natural polysaccharides can stimulate innate immunity by activating upstream immune cells, thereby regulating T cells and other adaptive immune pathways, and improving efficacy of immunotherapy (116–118). This affirms polysaccharides’ broad application in cancer treatment. For example, *Ganoderma lucidum* polysaccharides (GL-PS) represent an important bioactive component recognized as a natural source of immunomodulatory and anti-cancer compounds. Wang et al. (119) showed that GL-PS could increase the concentration of serum IL-2, TNF-α, and interferon-γ, thereby enhancing cytotoxic activity of NK and T cells, and promoting function of DCs, and ultimately inhibiting growth of gliomas and prolonging survival rates of rats. In addition, Bamodu et al. (120) demonstrated that *astragalus* polysaccharide (PG2) could significantly increase the rate of polarization of M1/M2 macrophages in non-small cell carcinoma (NSCLC) H441 and H1299 cells. Moreover, it promoted maturation of DC function and enhanced anti-cancer immune response mediated by T cells. In addition, it synergistically enhanced the anti-M2 anti-cancer effect of cisplatin and inhibited the growth of xenograft tumors in NSCLC mouse models. Chen et al. (121) studied the immunomodulatory activity of *Polygonatum sibiricum* polysaccharide (PSP) and elucidated its underlying mechanism of action by monitoring changes in immune organs, immune cells, and cytokines. Experimental results showed that PSP not only enhanced immune function of normal mice, but also participated in immunosuppressive protection of Cy-treated mice, which suggested its potential as an immunostimulant. Xu et al. (122) found that *Rehmannia glutinosa* polysaccharide (RGP) was a novel DC maturation reagent after analyzing its effect on activation of DCs as well as anti-cancer immune effect *in vivo*. RGP can used as an adjuvant for inducing activation of antigen-specific Th1 and CTL, as well as inhibiting growth of As-expressing tumors *in vivo*, including B16 melanoma and CT26 cancer cells.

Although natural polysaccharides exhibit immune properties, they are unstable in the body. Moreover, poor targeting and low bioavailability have hindered their clinical conversion. In order to overcome these shortcomings, researchers have developed strategies for stable delivery of polysaccharides to target sites and found that a combination of nano-carriers and polysaccharides can achieve excellent biocompatibility as well as reliable pharmaceutical protection. Consequently, several nanostructures have been designed to prolong the half-life of polysaccharides in the blood and promote their accumulation in immune organs, thereby enhancing immune activation. For example, Huang et al. (123) developed an RGP-based PEGylated nanoparticle (pRL), and found that *in vitro*, it was internalized into DCs through different endocytic pathways, a phenomenon that elevates cell proliferation and cytokine secretion. However, *in vivo*, pRL targets lymph nodes (LN) to present antigens, activates DCs in LN, and effectively induces effector T cell responses as well as powerful adaptive immune responses. On the other hand, Pang et al. (124) demonstrated that polysaccharides in natural herbs could be integrated into nanocomposites to improve efficacy of tumor treatment. Specifically, gold nanocomposite containing *Ganoderma lucidum* polysaccharide (GLP-Au) effectively induced activation of DCs. Moreover, chemotherapy drugs may cause damage to the immune system by reducing the percentage of CD4^+^/CD8^+^T lymphocytes. Results from a combined treatment, comprising Adriamycin, revealed that GLP-Au could reverse the decline in the number of CD4^+^/CD8^+^T cells, and generate a strong inhibitory effect on 4T1 tumor growth as well as lung metastasis. Another research group prepared AuNP-APS nanocomposites (APS-AuNP), loaded and presented *Astragalus* polysaccharide (APS) on the surface of AuNP, to promote direct interaction between APS and toll-like receptor 4 (TLR4) (125). Results showed that APS-AuNP could promote proliferation of T cells by inducing maturation of DCs and enhancing its killing effect on 4T1 tumor cells. In addition, APS-AuNP exhibited a strong ability to increase the number of CD4^+^/CD8^+^T lymphocytes.

Furthermore, Guo et al. (126) exploited the advantages of *Angelica* polysaccharides and natural Chinese medicine *Curcumin* to design functionalized nano-particle micelles wrapped in red blood cell membranes. Their administration in mice showed that immunomodulatory effect upregulated IL-12, TNF-α, and IFN-γ expression, and caused a 1.9-fold increase in CD8^+^ T cell infiltration, relative to the normal saline group. On the other hand, Yang et al. (127) designed a simple amphiphilic micellar nanocarrier for simultaneous delivery of *Lepidium meyenii* Walp. (maca) polysaccharide (MP) and *Chloroquine* (CQ) to TAMs. Their results indicated that MP could not only be used as a hydrophilic fragment for construction of micelles with TAM targeting, but also as an immunomodulator of TME. First, results from a comparison with free drug showed that the co-delivery system exhibited a TAM targeting ability. Second, construction of 4T1-M2 macrophage co-culture and 4T1 tumor xenotransplantation mouse models revealed that the co-delivery vector could exert simultaneous immunomodulatory effects of CQ and MP, thereby affirming the synergistic effect in cancer immunotherapy. Inspired by the excellent efficacy of immunomodulatory activity of *Angelica* polysaccharide (AP), Wang et al. (128) constructed an enzyme-sensitive tumor-targeted nano-drug delivery system (AP-PP-DOX, PP stands for peptide), and found that AP not only served as a carrier for targeted drug delivery to tumor tissues, but also acted as an effector for improving tumor microenvironment as well as enhancement of immune function. Consequently, the system produced a synergistic anti-tumor effect with chemotherapeutic drugs. In the presence of matrix metalloproteinase 2(MMP2), DOX and AP were quickly released from AP-PP-DOX, with the released DOX showing excellent anti-tumor efficacy. Moreover, the released AP upregulated IL-2 expression, but downregulated IL-10, indicating that it has potential to restore the Th1/Th2 immune balance in the tumor microenvironment.

## The Application of Nanoparticle-Based Adoptive T-Cell Transfer in Immunotherapy

Although adoptive transfer of therapeutic cells to cancer patients is demonstrated with great success, there are some potential issues, including poor tumor infiltration, and *in vivo* poor functional sustainability, as well as poor tumor-targeting efficiency (129). The explosion of emerging nanotechnology has improved ACT for cancer treatment. The *ex vivo* expansion and stimulation of T cells with natural APCs is time-consuming and exhibits poor reproducibility. To improve the expanding efficiency of anti-tumor T cells, an important strategy is to construct artificial antigen-presenting cells (aAPCs). Zhang (130) et al. developed biomimetic magnetosomes as multifunctional aAPCs to retain the biofilm properties of native APCs. Briefly, magnetic nanoclusters (MNCs) with superparamagnetic and magnetic responses were coated with leukocyte membrane fragments (LMNCs) and then modified with T-cell stimuli through copper-free click chemistry. These nano-aAPCs not only exhibited high performance for antigen-specific cytotoxic T cell (CTL) expansion and stimulation, but also effectively guide reperfused CTLs into tumor tissue visually by magnetic resonance imaging (MRI) and magnetic control. The persisting T cells were able to delay tumor growth, as demonstrated in the C57BL/6 mice model. Schmid’s research group prepared polymer nanoparticles that encapsulate CTLA-4 siRNA (131). After T cells took up the nanoparticles, the expression of CTLA-4 was down-regulated, resulting in enhanced T cell activation and proliferation. Injecting these nanoparticles into tumor-bearing mice increased the number of activated CD8^+^ T cells within the tumor. At the same time, some immunomodulatory drugs are also delivered to T cells in the body by this method, which can directly regulate their anti-tumor immune responses to suppress tumors.

Lipopolysaccharide (LPS) is a major component of the cell wall of Gram-negative bacteria and is a ligand for Toll-like receptor 4, which induces DC maturation, thereby inducing T cell responses. Demento (132) *et al.* designed a lipopolysaccharide-modified poly(lactic-co-glycolic) (PLGA) nanoparticle. The nanoparticle not only can initiate antigen presentation by dendritic cells, but also can present antigens or release cytokines in the vicinity of T cells. Experiments have shown that the nanoparticles can effectively induce humoral and cellular immunity in mice.

## The Application of Stimuli-Responsive Nanoparticles in Immunotherapy

Tumor tissues have specific microenvironments that are different from those of normal tissues, such as acidity, hypoxia, and overexpression of enzymes, among others (133). The biggest obstacle to anti-tumor immunotherapy is the low immunogenicity of tumor cells and immunosuppression caused by the tumor microenvironment (TME) (134). Therefore, remodeling the microenvironment, promoting immune cell infiltration, as well as inhibiting tumor angiogenesis and tumor metastasis are imperative to improving efficacy of anti-tumor drugs (135, 136). Constructing a multifunctional nano-drug delivery system can improve specific accumulation of drugs in tumor tissues, improve cellular uptake and release behavior of drugs, and regulate the tumor immunosuppressive microenvironment, thereby improving immune synergistic therapy and efficacy of tumor treatment (137, 138).

In recent years, researchers have designed a variety of microenvironment-responsive nano-delivery systems based on the microenvironment characteristics of tumor tissues. Notably, these systems can specifically respond to stimulation signals from the tumor’s extracellular or intracellular microenvironment, and enhance drug retention, accumulation, penetration, and tumor cell uptake at tumor sites. Previous studies overexpressed matrix metalloproteinases (MMPs) in solid tumor tissues to develop a tumor tissue MMP-2-responsive nano-drug delivery system that co-delivers PD-L1 antibody and photosensitizer indocyanine green (ICG) (139, 140). Particularly, ICG produces copious amounts of reactive oxygen species under near-infrared laser irradiation, which promotes release of tumor-associated antigens from tumor cells and infiltration of cytotoxic T cells in tumor tissues. The authors found that the developed nano drug delivery system effectively inhibited about 85% of tumor growth, improved the immunosuppressive microenvironment of 4T1 tumors, and significantly improved therapeutic efficacy of *in situ* tumors. Zhou et al. (141) exploited the characteristics of the tumor’s acidic microenvironment to construct a prodrug nanovesicle activated by the tumor’s extracellular acidic environment, and co-delivered oxaliplatin (OXA) prodrug as well as PEGylated photosensitizer (PS). Their results indicated that the nanovesicles promoted high-efficiency accumulation and penetration of drugs in tumor sites, and synergistically interacted to promote cell immunogenic death and activate the body’s immune response. Moreover, the authors observed significant differences in glutathione (GSH) concentrations between tumor and normal cells, which affirms effective drug release in tumor cells (142). Based on these results, the researchers developed a dual response nano-drug delivery system to co-deliver OXA and NLG919 (143), which responded to both a micro-acid environment outside the tumor cells and a reducing micro-environment inside the tumor cells. Results indicated that OXA could induce immunogenic death of tumor cells, while NLG919 inhibited activity of indoleamine 2,3-dioxygenase 1(IDO-1) in tumor cells, thereby overcoming the tumor immunosuppressive microenvironment. Results from tumor suppression experiments, using a tumor-bearing mouse model, showed that the inhibitory rate of the nano-drug delivery system on 4T1 *in situ* tumor growth and lung metastasis was 2.1 times and 2.0 times that of OXA and NLG919, respectively.

Han et al. (144) remodeled the tumor microenvironment by reversing the TAM phenotype for tumor immunotherapy, then prepared PLGA nanoparticles encapsulating baicalin and melanoma antigen Hgp peptide fragments. They further loaded the nanoparticles with CpG fragments, then used the conjugated M2pep (an M2-like macrophage binding peptide) and α-peptide (a scavenger receptor B type1 targeting peptide) on their surface to prepare novel nanocomposites. Results showed that the nanocomposites could be effectively ingested both inside and outside the body, while the acidic lysosomal environment triggered disintegration of polydopamine from the surface of the nanoparticles, thereby resulting in release of the payload. Furthermore, the nanocomposite promoted the delivery of baicalin, antigens, and immune stimuli to M2-like TAM, polarized and reversed the M2-like TAM phenotype, and remodeled the tumor microenvironment, thereby causing the death of tumor cells. The tumor microenvironment contains tumor-associated fibroblasts (CAF), which play an important role in formation of the tumor microenvironment and its associated immunosuppression (145). Zheng et al. (146) constructed a gold nanoplatform, loaded with doxorubicin (DOX), and found that the photothermal effect mediated by the gold shell could remodel the tumor microenvironment by reducing CAF and promoting release of DOX from nanoparticles. Their experimental results further showed that the nano-platform could actively target and recognize tumor cells, and hence activate immune response. At the same time, results from a BT474 nude mouse model revealed that the nano platform particles exhibited excellent anti-tumor efficacy. On the other hand, Shen et al. (147) constructed bifunctional liposomes (aNLG/Oxa(IV) by self-assembly of oxaliplatin prodrug (Oxa(IV) conjugated phospholipid and alkylated NLG919 (aNLG), an IDO1 inhibitor, and found a synergistic therapeutic effect on subcutaneous and orthotopic colorectal tumor models. This phenomenon was attributed to the aNLG/Oxa(IV) lip inducing effective immunogenic death of tumor cells, although the immunosuppressive TME could be reversed by inactivating IDO-1. Moreover, PTX is an important chemotherapeutic drug for clinical treatment of solid tumors, with studies showing that it causes the death of tumor cells and produces tumor-associated antigens (TAAs). Particularly, R837 has been shown to bind to the Toll-like receptors (TLRs) of DC thereby initiating TAA-specific T cell immune response, and upregulating TAA (148). Kang et al. (149) used iRGD peptide derivatives to co-assemble Paclitaxel (PTX) LPs and Imiquimod (R837) LPs to form tumor-targeted nano-assemblies (NAs), and found that NAs could proportionately release drugs to the tumor site and effectively inhibit tumor growth. Notably, therapeutic efficacy of tumors is achieved by inducing cell apoptosis and inhibiting angiogenesis, while enhancing the specific anti-tumor immune response can successfully prevent tumor recurrence.

## Conclusion and Outlook

Although tumor immunotherapy has obvious advantages with regard to inhibiting tumor growth, metastasis, and recurrence, its development and application are limited by low immune response, poor tumor targeting, and severe side effects. Applying the advantages of nanotechnology to tumor immunotherapy can effectively protect drugs from accidental degradation and help achieve long-term circulation in the blood as well as target tumor sites. To address numerous challenges limiting the current tumor immunotherapy strategies, there is a need to modify and transform the structure of nanoparticles. In addition, realizing the controllable time and space of immunotherapy is vital for accurate activation of immune response and alleviation of immune suppression. Therefore, nanotechnology in combination with tumor immunotherapy has an enormous potential for clinical transformation.

Although numerous *in vivo* studies have reported encouraging anti-tumor efficacies, clinical transformation of nano-delivery systems for tumor immunotherapy is still in its infancy, and many issues need to be considered. First, the preparation process for a nano-delivery system is usually overly complicated while industrial production is difficult. Therefore, the development and optimization of an effective delivery system is needed for effective application. Second, the nano-delivery system needs to be fully characterized, and its underlying delivery mechanism comprehensively elucidated. This is imperative to improve the therapeutic effect and assess the safety risks *in vivo*. Third, stringent quality control standards are needed to ensure stability and reproducibility of the process.

In the future, the development of a nano-delivery system for tumor immunotherapy is expected to focus on two main goals, namely enhancing anti-tumor immune response and overcoming immunosuppression. To this end, several key considerations should be considered to ensure accelerated clinical conversion. First, further research on tumor microenvironment and metabolic pathways should be devoted to improving cancer immunotherapy by regulating tumor microenvironment and metabolism. Second, there is a need to develop nano-delivery systems with imaging functions and stimulus activation characteristics, to specifically respond to the tumor microenvironment. Notably, the development of nanomedicine should focus on integrating diagnosis and treatment strategies for tumor immunotherapy. Third, there is a need for interdisciplinary development, comprising expertise from experts across immunology, materials science, pharmacology, and other fields, which will help in discovering new immune targets and pathways to facilitate development of new drug delivery systems.

## Author Contributions

LZ and MZ contributed equally to this work, drafted the manuscript. YX and PL collected important background information. CL analyzed data and document. XX reviewed and revised manuscripts. All authors contributed to the article and approved the submitted version.

## Funding

This work was supported by the Natural Science Foundation of Changsha (NO. kq2014090), the Natural Science Foundation of Hunan Province (NO.2020JJ5417), and the National Science Foundation of China (NO. 81573621).

## Conflict of Interest

The authors declare that the research was conducted in the absence of any commercial or financial relationships that could be construed as a potential conflict of interest.

## Publisher’s Note

All claims expressed in this article are solely those of the authors and do not necessarily represent those of their affiliated organizations, or those of the publisher, the editors and the reviewers. Any product that may be evaluated in this article, or claim that may be made by its manufacturer, is not guaranteed or endorsed by the publisher.
